# An Updated Review on Head and Neck Cancer Treatment with Radiation Therapy

**DOI:** 10.3390/cancers13194912

**Published:** 2021-09-30

**Authors:** Garrett Anderson, Maryam Ebadi, Kim Vo, Jennifer Novak, Ameish Govindarajan, Arya Amini

**Affiliations:** 1 Division of Nuclear Medicine, Department of Radiology, Loma Linda University, Loma Linda, CA 92350, USA; garrett.g.anderson.2020@gmail.com; 2 Department of Internal Medicine, Louis A. Weiss Memorial Hospital, Chicago, IL 60640, USA; mebadi@umn.edu; 3 College of Osteopathic Medicine of the Pacific, Western University of Health Sciences, Pomona, CA 91766, USA; kimvo.darling@gmail.com; 4 Department of Radiation Oncology, City of Hope National Medical Center, Duarte, CA 91010, USA; jnovak@coh.org; 5 Department of Internal Medicine, UCLA-Kern Medical Center, Bakersfield, CA 93306, USA; ameishgovindarajnmd@gmail.com

**Keywords:** oral cavity cancer, oropharynx, larynx, hypopharynx, head and neck cancer, squamous cell carcinoma, adjuvant radiation therapy, postoperative radiation therapy

## Abstract

**Simple Summary:**

The mainstay of treatment for locoregionally advanced head and neck squamous cell carcinoma (HNSCC) is either surgery followed by adjuvant radiation therapy or definitive concurrent chemoradiation (CRT) reserving surgery as salvage therapy, referred to as the organ-preservation approach. Head and neck cancer treatment requires a multidisciplinary approach with medical, surgical, and radiation oncology, pathology, radiology, and supportive services including physical and occupational therapy, speech and swallow therapy, and nutrition. The field has rapidly evolved with rising rates of HPV positive oropharyngeal cancers leading to treatment de-escalation studies that are currently ongoing. Additionally, multiple trials are ongoing to evaluate the role of novel agents including immune checkpoint inhibitors, less invasive surgical approaches, and radiation field and dose reductions in order to maintain effective tumor control while improving quality of life outcomes for our head and neck cancer patients.

**Abstract:**

The complexity of head and neck cancers (HNC) mandates a multidisciplinary approach and radiation therapy (RT) plays a critical role in the optimal management of patients with HNC, either as frontline or adjuvant treatment postoperatively. The advent of both definitive and post-operative RT has significantly improved the outcomes of patients with HNC. Herein, we discuss the role of postoperative RT in different subtypes of HNC, its side effects, and the importance of surveillance. The treatment regions discussed in this paper are the oral cavity, nasopharynx, paranasal sinus cavity, oropharynx, larynx and hypopharynx. Multiple studies that demonstrate the importance of definitive and/or postoperative RT, which led to an improved outlook of survival for HNC patients will be discussed.

## 1. Introduction

**Figure 1:**
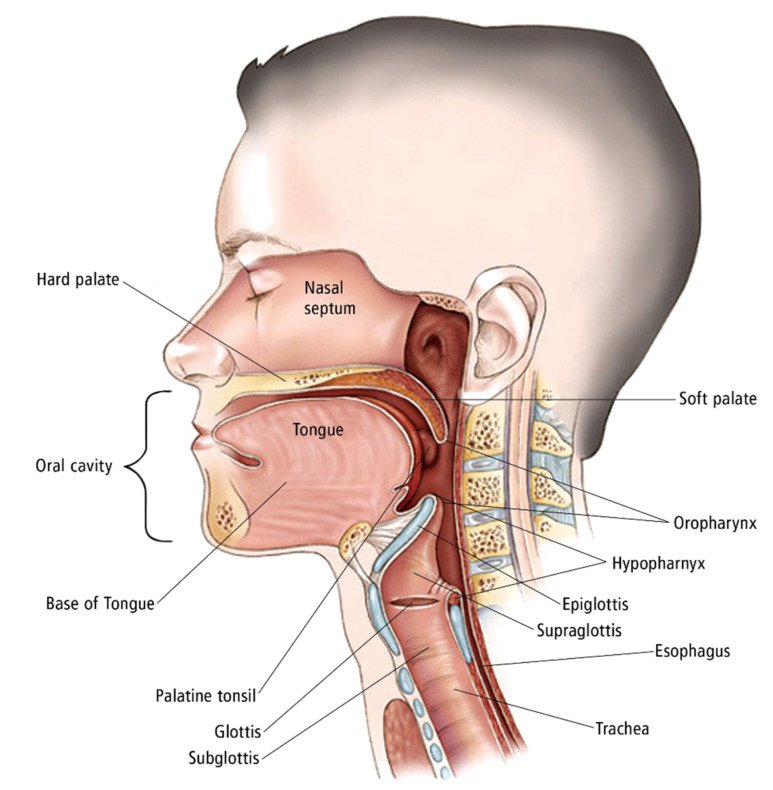
Illustration of the head and neck. The oral cavity includes the mucosal surface of the lips, the anterior two-thirds of the tongue, buccal mucosa, retromolar trigone and the hard palate. The oropharynx just posterior to the oral cavity includes the soft palate, the palatine tonsils, and base of the tongue. The nasopharynx located superiorly is bordered by the nasal cavity, clivus, soft palate, and sphenoid. The larynx includes the supraglottis, glottis, and subglottis. The hypopharynx just posterior to the larynx includes the following subsites: piriform sinus, postcricoid space, and posterior pharyngeal wall. Additional sites of the head and neck including the nasal cavity and paranasal sinuses (not imaged). Copyright 2005-2011 American Society of Clinical Oncology. Robert Morreale/Visual Explanations, LLC.

The mainstay of treatment for locoregionally advanced head and neck squamous cell carcinoma (HNSCC) is either surgery followed by adjuvant radiation therapy (aRT) or definitive concurrent chemoradiation (CRT) reserving surgery as salvage therapy, referred to as the organ-preservation approach. [1]. Head and Neck cancers are complex with multiple subsites (Figure 1) require different oncologic approaches for cure and therefore require a multidisciplinary approach with trimodality therapy including the need for support teams including speech and swallow therapy, physical therapy, occupational therapy, smoking cessation programs, and nutrition. This paper aims to help explain each nuisance for aRT head and neck cancers.

## 2. Oral Cavity

Cancers that arise in the oral cavity provide a unique clinical challenge for radiation oncologists. Oral cavity cancers (OCC) are commonly caused by smoking, betel nut use, alcohol, poor oral hygiene, and presentation of oral leukoplakia or erythroplakia. OCCs are managed by upfront surgery if resectable followed by adjuvant therapy including radiation and potentially chemotherapy if indicated. [1]. In the oral cavity, stage I and II (early tumors) are treated with primary surgery or definitive RT, while stage III and IV (locoregionally advanced) are treated with surgery followed by aRT with or without CRT. Radiation doses for OCC in the postoperative setting ranges from 70 Gy for gross disease, 60–66 Gy for high-risk regions, and 50–54 Gy to cover low-risk areas subject to microscopic spread. In the definitive setting, doses of 66–70 Gy are typically used with chemotherapy or alternative fractionation schemas including hypofractionation or hyperfractionation for patients receiving radiation alone.

In a multicenter randomized controlled trial (RTC), Overgaard et al. [2]. discussed the benefits of increasing the total number of fractions a week from five to six, for increased primary tumor control. Patients in the six fractions a week instead of five had better primary tumor control at 76% vs. 64% (hazard ratio [HR] 0.63, 95% CI 0.49–0.83; *p* = 0.004). Rosenthal et al. [3]. conducted a phase III RCT on 264 patients with stage III-IV SCC of the oral cavity, oropharynx, or hypopharynx. Low-risk regions were treated with RT at doses of 57.6 Gy or 63 Gy, while high-risk regions were randomized to receive 63 or 68.4 Gy, over 1.8 Gy per fraction. Overall survival (OS) rates for 5- and 10-year marks were found to be 32% and 20%, respectively. The study has found that increasing the dose from 57.5 to 68.4 Gy without chemotherapy does not improve tumor control. However, treatment package time (TPT) shorter than 85 days demonstrates better locoregional control compared to >85 days for dose levels >60 Gy. Shortening TPT in the study improved cancer specific survival (CSS), locoregional control (LRC) and OS for HNSCC, independently of the total RT dose delivered.

RT to the oral cavity and bilateral neck can be very toxic. One out of every five patients will develop oral mucositis. [4]. Mucositis is a dose limiting toxicity that is thought to affect patients in different severity due to each patient’s unique oral microbiome. When patients develop oral mucositis, a burning discomfort accompanied by pain from the inflammation of the mucosal lining can be felt. These sensations can make it very difficult for patients to eat, drink, talk and swallow if they are severe. Due to this, mucositis needs to be monitored closely to ensure that patients will not be discontinued from RT or end up hospitalized.

Exposure of the tongue can also lead to partial muscle paralysis, making it difficult for the patient to speak properly when dosed above 30 Gy. Over time, this becomes problematic when patients lose weight from not consuming food. If left untreated there is a potential need for intervention with a feeding tube to prevent hospitalizations and breaks in treatment. Taste disturbance (dysgeusia) is another notable symptom in patients who received radiation therapy in the oral cavity. [5,6]. Direct exposure of the oral cavity to radiation damages taste buds, causing hypogeusia or dysgeusia in patients, affecting their quality of life. [7]. There are limited options to prevent dysgeusia; however, many patients do regain taste after completing the treatment. Xerostomia or dry mouth develops into dysgeusia and dysphagia overtime if the condition goes unaddressed medically. [5,8]. Xerostomia is largely dependent on the radiation dose to the parotid and submandibular glands. Radiation associated damage to the salivary glands are reversible at doses under 30 Gy, showing increased salivation somewhat near pretreatment levels. [5,7]. However, in higher doses, around 75 Gy, there is significant destruction of the salivary glands and decreased salivation levels [9]. The use of intensity-modulated radiation therapy (IMRT) has significantly reduced the risk of xerostomia for these patients. These advances to specifically target the primary lesion with IMRT, allows radiation oncologists to avoid irradiating other tissues not directly adjacent to the treatment lesion to avoid physiological “stunning” by radiation therapy.

Whitmore et al. [10]. discusses how an established oral microbiome can lead to opportunistic infections. *Porphyromonas gingivalis* and *Fusobacterium nucleatum* are the two main bacterial species that lead to periodontal disease and develop chronic inflammatory conditions that lead to the development of OCC through their molecular interactions in the oral cavity. The addition of oral opportunistic infections while patients are dealing with RT-related side effects can be hazardous to the treatment recovery process. RT-induced osteonecrosis can also manifest in the surrounding osseous structures that absorb a larger than normal radiation dose [11]. The loss of bone density due to osteonecrosis can leave many patients even more vulnerable to fractures, further limiting their quality of life. Although the primary site of RT-induced osteonecrosis is the mandible, due to the low blood perfusion compared to the other osseous structures in the oral cavity, the surrounding structures may also be at risk following RT. In extreme cases, this necessitates the complete removal of necrotic osseous tissues. An in-depth understanding of the proper beam arrangements, determined by target volumes, is vital to ensure that proper dosage is delivered to the OCC while attempting to reduce the overall absorbed radiation dose to the osseous structures in the oral cavity [12]. With proper patient positioning, regions of osseous and glandular tissue can be spared to help maintain the quality of life for patients following adjuvant radiation therapy.

RT-associated toxicities, esp. aspiration pneumonia, can be fatal. Aspiration pneumonia occurs due to swallowing dysfunction caused by xerostomia, thickened oral secretions, and mucositis in the acute setting and radiation-induced fibrosis in the chronic setting. [13]. In 5658 patients who received curative-intent RT for head and neck SCC, 90-day mortality was seen in 3.6% of the patients, 0.5% of which were due to aspiration pneumonia [14]. In another study, 60-day mortality due to aspiration pneumonia occurred in 1.2% of 592 head and neck cancer patients treated with chemoradiation (CRT) [15].

## 3. Oropharynx

Cancers of the oropharynx include the palatine tonsils, lingual tonsils/base of tongue, soft palate, and adjacent pharyngeal walls. The tonsils are a collection of lymphoid tissues that are part of the aerodigestive tract that regulate the initial part of the immune function following the ingestion of food [14]. Oropharyngeal SCC (OPSCC) is known to be caused through lifestyle factors linked to high levels of tobacco and alcohol use. Human papillomavirus (HPV) is a prognostic marker in OPSCCrecent [15,16]. and tumor HPV positivity is associated with substantially higher cure rates and improved survival. HPV- RT is similar to other head and neck tumor types following:45–50 Gy for microscopic disease, 56–60 Gy for high-risk areas, and 70 Gy for gross disease [17]. RT is typically delivered with concurrent cisplatin given every 3 weeks at 100 mg/m^2^ or weekly at 40 mg/m^2^. Two phase II/III randomized controlled trials are attempting to demonstrate that nivolumab is non-inferior to standard of care cisplatin in HPV oropharyngeal cancer. The NRG HN005 study is a three-armed study comparing the current standard of care (70 Gy with cisplatin) to either lower dose RT with cisplatin or RT with nivolumab. [18]. As of now, the standard remains cisplatin-based chemotherapy as two randomized trials showed inferior outcomes with cetuximab [19].

Currently, the standard treatment for all oropharyngeal cancers is similar, regardless of the tumor HPV status. However, multiple recent studies evaluated the possibility of de-escalating therapy in HPV-positive OPSCC to reduce treatment-related toxicities, while delivering effective curative intent treatment [20]. All these studies have concluded that 2-year or 3-year PFS/OS are comparable to standard therapy.

The phase II Quarterback trial compared standard dose CRT (sdCRT) with reduced-dose CRT (rdCRT) after induction chemotherapy (IC) with docetaxel, cisplatin and fluorouracil (3 cycles) in previously untreated, locally advanced HPV+ OPSCC patients with a ≤20 pack years smoking history. Those who had complete clinical response (CR) to IC were then randomized 1:2 to sdCRT (7000 cGy) or rdCRT (5600 cGy) with weekly carboplatin. Three-year PFS/OS was similar between the sdCRT (n = 8) and rdCRT (n = 12) groups (87.5% vs. 83.3%, respectively). [21]. However, the study was limited by small sample size. The single-arm phase II E1308 trial investigated the clinical outcomes of patients receiving reduced-dose RT (54 Gy) with weekly cetuximab 250 mg/m^2^. Eligible patients had respectable HPV+, stage III or IV OPSCC and had achieved a CR after IC. Induction chemotherapy included cisplatin, paclitaxel and cetuximab. 51 patients were in CR after IC and received rdCRT. 2-year PFS and OS were 80% and 94%, respectively [22]. Another phase II clinical trial (NCT02048020/NCT01716195) also investigated the outcomes of rdCRT in patients with HPV+ OPSCC. All patients received IC with 2 cycles of paclitaxel and carboplatin. Patients (n=24) who achieved CR or partial response (PR) received rd-CRT (RT: 54 Gy with weekly paclitaxel), and those who did not achieve a PR or CR (n = 20) received rdCRT, but at a higher dose (RT: 60 Gy with weekly paclitaxel). 2-year PFS and OS were 92% and 98%, respectively, in the entire study population. 39% experienced grade 3 toxicity, but no grade 4 toxicity was seen. The authors concluded that 15–20% reduction in RT dose was associated with high PFS and an improved toxicity profile compared to standard dose [23]. The phase II MC1273 trial investigated the role of adjuvant RT de-escalation after curative intent surgical resection and neck dissection. Inclusion criteria were HPV+ OPSCC with ≤10 pack-year smoking history, negative margins after surgery, and at least one pathologic risk factor (i.e., extranodal extension, lymphovascular or perineural invasion, ≥T3, ≥N2). Patients with (n = 43) vs. without (n = 37) extranodal extension received RT at 36 Gy vs. 30 Gy, both concurrent with docetaxel. 2-year PFS and OS in the whole study population was 91% and 99%, respectively. Toxicity and gastrostomy tube use rates were very low [24]. Grade ≥ 3 toxicity rates at pre-RT was 2.5%. All Grade ≥ 3 toxicities resolved by 6 months post-RT. The study concluded that aggressive dose de-escalation from 60 and 66 Gy to 30 and 36 Gy of aRT yielded comparable locoregional tumor control rates, with less toxicity. The EVADER study (CCTG HN.10.) is an ongoing phase II clinical trial evaluating the role of volume adjusted de-escalation radiotherapy in patients with low-risk HPV+ OPSCC with the goal to reduce short- and long-term treatment-related morbidity [25]. This is a single arm study, and the hypothesis is that by decreasing the regions of elective nodal irradiation, high disease control rates in patients with favorable prognosis HPV+ OSCC is maintained, while treatment-related toxicity is reduced, resulting in improved quality of life.

## 4. Nasopharynx

Nasopharyngeal carcinoma (NPC) is a distinct epithelial carcinoma of the head and neck, commonly arising from the mucosal lining of the fossa of Rosenmüller. It is more prevalent in Asia and less common in the US and Western Europe. The incidence has been declining with 129,000 cases diagnosed in 2018. Despite a decrease in the overall incidence, new cases in adolescents and younger adults have been increasing [26]. It is twice more common in men, for unknown reasons. There are three pathological subtypes: keratinizing squamous, non-keratinizing, and basaloid nasopharyngeal carcinoma. Non-keratinizing NPC, prevalent in the 40–59 years age group, is the most common subtype and consists of two subgroups: differentiated and undifferentiated. Host genetics, Epstein–Barr virus (EBV), and environmental factors including alcohol, smoking, and nitrosamines play a role in NPC etiology [27,28].

Since NPC is highly sensitive to RT and chemotherapy, the standard treatment is RT with or without chemotherapy depending on the stage of disease. CRT is the standard treatment for locoregionally advanced and non-metastatic NPC [29]. Stage I NPCs generally are managed with RT alone, with good locoregional control and a 5-year OS of 90%. [30]. RT doses range from 45–50.4 Gy for microscopic disease, approximately 59–60 Gy for high-risk regions, and 69–70 Gy for gross disease in the neck, all in single daily fractions of 2 Gy, 5 days a week for 6–7 weeks using intensity-modulated radiation therapy (IMRT). Currently, there is no clear role for adjuvant chemotherapy given mixed results and its efficacy and selection of chemotherapy are being evaluated in the NRG HN001 study based on EBV levels [31]. The role of IC is also somewhat debated. In a multicenter phase III RCT in 480 evaluable adults with previously untreated, stage III–IVB NPC, Sun et al. [32]. compared the outcomes of IC before CRT with CRT alone. CRT consisted of three cycles of 100 mg/m² cisplatin every 3 weeks and RT using IMRT. Both groups received RT at a median dose of 70 Gy. IC comprised cisplatin, fluorouracil, and docetaxel. Three-year progression free survival (PFS), OS and disease-free survival (DFS) were significantly higher in the group who received chemotherapy before CCRT (80% vs. 72%, *p* = 0.034; 95% vs. 86%, *p* = 0.029 and 90% vs. 83%, *p* = 00.031, respectively). However, locoregional failure-free survival was not significantly different between the groups (95% vs. 89%, *p* = 00.12). As expected, chemotherapy-related Grade 3 and 4 adverse events were higher in those who received IC; neutropenia 42% vs. 7%, leukopenia 41% vs. 17%, and stomatitis 41% vs. 35%. Long-term follow-up is needed to better characterize the long-lasting efficacy and toxicity.

The role of adjuvant chemotherapy is further being delineated as mentioned above in the ongoing NRG trial. Using the data from a prospective multicenter EBV DNA screening cohort, Hui et al. [33]. aimed to develop a validation tool to identify patients who would benefit from adjuvant therapy in NPC after curative RT or CRT. The study enrolled NPC patients between 2006–2015. Considering that post-RT circulating plasma EBV DNA is associated with minimal residual disease and predicts higher relapse rates and worse survival independently of disease stage, the authors hypothesized that EBV DNA level would improve the prognostic value of TNM staging by better identifying higher-risk patients who would benefit from adjuvant therapy vs. lower-risk patients who could be spared from unnecessary adjuvant therapy and its toxicity [34].

## 5. Larynx 

Laryngeal squamous cell carcinoma is the second most common head and neck cancer [35]. This is due to the pathogenesis being linked to smoking. The larynx is part of the respiratory system originating at the connection of the epiglottis and esophagus. The largest concern for laryngeal cancer RT patients that needs to be monitored are occlusion/protection of the airway during swallowing, phonation, and breathing which require a multidisciplinary team for treatment [36,37]. Early-stage laryngeal cancer can be managed by single modality RT without surgical resection. More advanced laryngeal cancers are typically managed with CRT for voice preservation [38]. In T4 tumors with thyroid cartilage involvement, upfront surgery followed by adjuvant treatment is often recommended.

Standard radiation therapy for T1-T2 glottic cancers follows a hypofractionated dosing scheme for a total of 63-65 Gy [36]. In locally advanced cases undergoing concurrent CRT, RT dosing is like other primary head and neck sites discussed earlier. Forastiere et al [37]. discusses several phase III trials for laryngeal cancer supporting both hyperfractionation and accelerated fraction treatments which demonstrate a 10–15% in LRC of the primary tumor.

## 6. Hypopharynx 

Cancers of the hypopharynx originate at the pharyngoepiglottic fold and extend to the inferior aspect of the cricoid cartilage. Its margins are marked anteriorly by the posterior cricoid mucosa and the posterior cricoarytenoid muscle, posteriorly it reaches the mucosal wall including the middle and inferior constrictor muscles. Hypopharyngeal squamous cell carcinoma is typically diagnosed in the later stages of cancer (III-IV) due to the lack of clinical presentation until a bulk tumor volume can be palpated during swallowing [38].

RT for hypopharyngeal SCC follows a dosing scheme of 60–72 Gy with standard fractions of 2 Gy/day for seven weeks and is typically given with concurrent platinum-based chemotherapy [38]. Treatment areas should focus on the primary lesion and surrounding cervical lymph nodes. If the primary lesion is located adjacent to the spinal cord, it is cautioned when dosing treatments of over 40 Gy to minimize radiation exposure to the spinal cord; this can be minimized with IMRT [39].

## 7. Nasal Cavity/Paranasal Sinus 

Sinonasal tumors are rare accounting for 3–5% of all head and neck carcinomas. The mean age of diagnosis is 62 years, affecting more males than females (2:1 ratio) [40]. More than 80% of the cases are observed in Caucasians. Cigarette and industrial agents such as thorium dioxide, isopropyl oils, lacquer paints, solder and welding materials, wood dust, as well as radium watch-dial paint are known risk factors. SCC is the most common subtype accounting for 50% of all tumors. Less common types include adenocarcinoma, adenoid cystic carcinoma, melanoma, inverting papilloma, esthesioneuroblastoma, midline granuloma, lymphoma, and sarcoma. [40,41].

The mainstay of treatment for sinonasal malignancies is surgical resection followed by aRT with or without chemotherapy [42]. Among head and neck cancers, malignancies in the paranasal sinuses are associated with the highest local recurrence (up to 61%). The maxillary sinus is the most common site of origin for paranasal sinus malignancy. However, other sinuses (i.e., ethmoid, frontal, and sphenoid) can also be involved [43].

### 7.1. Sinonasal Squamous Cell Carcinoma

The mainstay of treatment for all locoregionally advanced head and neck SCCs is either surgery followed by aRT or upfront CRT, reserving surgery as salvage therapy [44]. In primary paranasal sinus tumors, complete resection of the tumor should be first attempted. In a RCT comparing the outcome of surgery followed by aRT with CRT in 119 patients with stage III/IV respectable head and neck SCC with 13 years of follow up, the two treatment modalities achieved similar OS and disease-specific survival (DSS). However, patients with primary paranasal sinus (particularly maxillary sinus) SCC who received surgery and aRT had a 5-year DSS rate of 71% compared to 0% in those who received CRT (*p* = 0.05) [45].

Goel et al. [42]. retrospectively studied 2267 patients with non-metastatic sinonasal SCC treated with surgery followed by aRT using the National Cancer Database (NCDB) over 10 years (2004–2014). Most patients received RT through IMRT (54.2%); 35% received 50–59.99 Gy, 36% received 60-65.99 Gy, and the remaining received higher doses. The median duration of diagnosis to surgery (DTS) was 32 days, surgery to radiation (SRT) 49 days, and radiation duration (RTD) 47 days. Delays in surgery or RT and the duration of RT treatment resulted in decreased OS. SRT longer than 64 days and RTD longer than 51 days led to significantly worse OS. Xiao et al. [46]. used the NCB to compare the outcomes of patients with nasal cavity or paranasal sinus SCC who received either endoscopic (n = 168) or open surgery (n = 168). Those who received endoscopic treatment had a significantly shorter postoperative time to aRT (PTTR) (51.2 vs. 58.4 days, *p* = 0.02) resulting in improved 3-year OS. PTTR shorter than 49 days was associated with significantly better OS (74.2% vs. 61.4%, *p* = 0.04) and was a strong predictor of OS.

SCC of the nasal vestibule and pyramid is a rare entity and the optimal treatment is controversial. Early-stage disease is typically treated with surgery or RT alone with Comparable outcomes, whereas a multimodality approach is used to treat advanced stage diseases [47]. Lambertoni et al. [48]. retrospectively studied 45 patients with SCC of the nasal vestibule and pyramid. All patients underwent surgical excision. Four patients with locally advanced high-grade tumors, lymphovascular or perineural invasion received post-operative RT to the primary site through IMRT (60 Gy in 30 fractions). Two patients received elective neck RT through IMRT (54 Gy in 30 fractions). Patients with positive surgical margins or nodal extracapsular spread in the neck received cisplatin-based chemotherapy concomitant with RT. 5- year OS and DFS were 82% and 62%, respectively. The authors recommended aRT and elective neck treatment in patients with advanced stage high-risk patients.

### 7.2. Sinonasal Adenocarcinoma

Sinonasal adenocarcinomas (SNAC) account for 10–20% of sinonasal malignancies, mainly originating from the respiratory epithelial or glandular cells of the sinonasal mucosa. Over 77% of the cases arise from the ethmoid sinus. [49]. Although the low-grade disease has a favorable prognosis, high-grade tumors have a poor prognosis with a 3-year OS of 20%. SNACs are classified as salivary (5–10%) and non-salivary. The non-salivary type is further categorized into intestinal-type (ITAC) and non-intestinal-type (NITAC) [49,50]. In both subtypes, surgery is the standard treatment modality for low-stage (pT1-2) and low-grade (papillary, colonic) tumors [48]. Surgery is followed by aRT in high-stage (pT3–4) and high-grade (solid/mucinous subtype, and/or involved margins) disease. A total RT dose of 50–70 Gy in 1.8–2 Gy fractions is delivered through IMRT. To determine the best treatment modality for early-stage (pT1-T2) disease, Turri-Zanoni et al. [51]. performed a retrospective study on 61 patients, 33 of whom had received endoscopic surgery alone and 28 patients had received aRT after surgery. Among those with high grade tumors (47 cases), aRT was associated with significantly better OS (90.5% vs 57.6%, *p* = 0.03) and relapse-free survival (RFS) (92.3% vs. 80.2%, *p* = 0.05) at a median follow-up of 5 years. ART had no survival benefit in low-grade SNAC.

### 7.3. Undifferentiated Carcinoma 

Sinonasal undifferentiated carcinoma (SNUC) is a rare, poorly differentiated, rapidly growing malignancy originating from the mucosa of the nasal cavity or paranasal sinuses. Patients typically present with locally advanced disease or distant metastases and only ~5% have T1 or T2 disease at the time of diagnosis. It is associated with poor outcomes with a 5 year-OS of 42%. Multimodal therapy is the mainstay of treatment [52]. Using the NCDB, Kuo et al. [51]. studied the role of combined modality treatment in 435 patients with SNUC by comparing the outcomes between patients who received optimal surgery and CRT, surgery + RT, RT alone or surgery alone. Treatment modality was a strong predictor of outcome in multivariate analysis. OS was significantly higher in the surgery + CRT group compared to surgery alone, surgery+ RT, and RT alone. However, there were no statistical differences between surgery + CRT and definitive CRT groups. In the surgery + CRT group, OS was similar between those who received induction CRT and adjuvant CRT (HR = 0.437, 95% CI = 0.138–1.39, *p* = 0.16). Khan et al. [53]. compared the outcomes of surgery and adjuvant CRT with definitive CRT in 304 patients with SNUC registered in the NCDB. 60% had advanced (stage III or IV) disease at the time of diagnosis. Among all patients, surgery + CRT led to significantly better 5-year OS compared to definitive CRT (55.8% vs. 42.6%, *p* = 0.007). However, among patients with advanced disease, there was no difference in survival between the 2 groups (*p* = 0.22). Surgery was beneficial only if resection with negative margins could reliably be performed. Amit et al. [54]. evaluated the possibility of using response to IC as a guide to definitive treatment in 95 patients who received curative intent treatment between 2001–2018. Among patients who achieved a partial or complete response after IC, 5-year DSS was significantly higher in those who received definitive CRT compared to those who received definitive surgery and postoperative RT or CRT (81% vs. 54%, respectively; *p* = 0.001). Among those with no response to IC, 5-year DSS was 0% in patients treated with CRT and 39% in those treated with surgery plus RT or CRT (adjusted HR 5.68, 95% CI: 2.89–9.36). The authors recommended using definitive CRT if the patient achieves partial or complete remission after IC and, if feasible, surgery in those who have no response to IC.

### 7.4. Esthesioneuroblastoma

Esthesioneuroblastoma (ENB) is a very rare neuroectodermal nasal cavity malignancy originating from the olfactory epithelium lining. ENB is associated with a high nodal recurrence of 30% [55]. The mainstay of treatment is surgical resection followed by aRT. Systemic chemotherapy is also given in patients with advanced disease [56]. Adjuvant RT has been associated with improved outcomes. In a retrospective review of 70 patients with stage T3 or T4, the addition of aRT to surgical resection significantly improved DSS by 10 years. [57,58].

The role of prophylactic neck irradiation in patients with N0 disease remains controversial. Jiang et al. [55]. studied 71 N0 patients who had received aRT. Radiation was given to the tumor bed alone in 86% and elective nodal irradiation (ENI) was performed in 31%. Most patients (92%) received surgical resection followed by RT. The remaining 8% were treated with either definitive RT or CRT. Elective node dissection was not performed. ENI significantly decreased regional nodal progression. However, it failed to improve OS or DFS. This might be due to the prolonged latency of nodal metastasis which is unique to ENB. Patients who received ENI achieved 100% 5-year locoregional control compared to 78% in those who did not undergo ENI. No patient in the ENI group experienced locoregional progression in 10 years of follow-up, while 37% of those who did not receive ENI progressed [59].

## 8. Future Directions

Therapy de-intensification for HPV+ OPSCC in appropriately selected patients has yielded promising results. Multiple clinical trials have shown that 2-year and 3-year PFS and OS are comparable to standard treatment with the benefit of reducing RT-related toxicities. Currently, de-intensification should only be performed in the setting of clinical trials, with the future goal to incorporate this approach into standard therapy. [60]. Patients with low recurrence risk (i.e., ≤10 pack-year smoking history, <T4, and<N2c disease) likely benefit from treatment de-escalation. Reduced RT dose also leads to significantly fewer swallowing and nutritional complications [22].

The other future direction is combining immunotherapy with RT. Preclinical studies have demonstrated that RT has substantial immunomodulatory effects, leading to growing interest in the potential synergy between immunotherapy and radiotherapy. It is proposed that RT can potentially augment anti-tumor immune response and immunotherapy may improve tumor-response to RT [61].

Pembrolizumab, a PD-1 inhibitor, has a proposed synergistic effect with RT. The phase II randomized trial, PembroRad, compared the outcomes of combining pembrolizumab with RT to concurrent cetuximab and RT. Patients with non-operable stage III-IVa-b SCC of oral cavity, oropharynx, hypopharynx and larynx and patients who were unfit for receiving high-dose cisplatin were randomized to either the cetuximab-RT arm (IMRT 69.96 Gy, cetuximab) or pembro-RT arm (same dose of RT and pembrolizumab). 65 patients were enrolled in the Cetux-RT arm and 66 patients in the Pembro-RT arm. Locoregional control was similar between the 2 arms (59% vs. 60% in the Cetux-RT arm and Pembro-RT arm, respectively). 2-year PFS and OS were comparable between the 2 groups (40% vs. 42% and 55% vs. 62% in the Cetux-RT and Pembro-RT arm, respectively). Patients in the Pembro-RT arm experienced less acute toxicity (74% vs. 92%) [62].

## 9. Conclusions

Radiation treatment for head and neck cancers remains one of the most challenging areas to treat for our patients, given the extent of side effects. In non-oral cavity patients undergoing definitive treatment with organ preservation, the standard of care remains concurrent chemoradiation. In the postoperative setting, radiation often is indicated based on risk factors with concurrent chemotherapy in patients with positive margins and/or ECE. Ongoing trials are looking to de-escalate treatment by reducing RT treatment volumes, RT dose, and potentially de-intensifying concurrent systemic therapy specifically for HPV positive oropharyngeal cancers. Additional trials are ongoing to evaluate the role of modern systemic agents including immune checkpoint inhibitors and their role with T in the treatment of head and neck cancer.
